# The extracellular microenvironment in immune dysregulation and inflammation in retinal disorders

**DOI:** 10.3389/fimmu.2023.1147037

**Published:** 2023-03-01

**Authors:** Fabiola Biasella, Karolina Plössl, Paul N. Baird, Bernhard H. F. Weber

**Affiliations:** ^1^ Institute of Human Genetics, University of Regensburg, Regensburg, Germany; ^2^ Department of Surgery, Ophthalmology, University of Melbourne, Melbourne, VIC, Australia; ^3^ Institute of Clinical Human Genetics, University Hospital Regensburg, Regensburg, Germany

**Keywords:** extracellular matrix, Bruch’s membrane, matricellular proteins, inflammation, retinal disease, age-related macular degeneration, Sorsby fundus dystrophy, TIMP3

## Abstract

Inherited retinal dystrophies (IRDs) as well as genetically complex retinal phenotypes represent a heterogenous group of ocular diseases, both on account of their phenotypic and genotypic characteristics. Therefore, overlaps in clinical features often complicate or even impede their correct clinical diagnosis. Deciphering the molecular basis of retinal diseases has not only aided in their disease classification but also helped in our understanding of how different molecular pathologies may share common pathomechanisms. In particular, these relate to dysregulation of two key processes that contribute to cellular integrity, namely extracellular matrix (ECM) homeostasis and inflammation. Pathological changes in the ECM of Bruch’s membrane have been described in both monogenic IRDs, such as Sorsby fundus dystrophy (SFD) and Doyne honeycomb retinal dystrophy (DHRD), as well as in the genetically complex age-related macular degeneration (AMD) or diabetic retinopathy (DR). Additionally, complement system dysfunction and distorted immune regulation may also represent a common connection between some IRDs and complex retinal degenerations. Through highlighting such overlaps in molecular pathology, this review aims to illuminate how inflammatory processes and ECM homeostasis are linked in the healthy retina and how their interplay may be disturbed in aging as well as in disease.

## 1 Introduction

Inherited retinal diseases (IRD) confer a diverse group of diseases presenting at varying ages in life. Many phenotypic features appear shared between the different IRDs that can make their clinical classification challenging although application of molecular diagnostic tools and testing can be applied to provide greater clinical certainty (1, 2). What has become clear in this process is that genetic heterogeneity exists between the majority of IRDs, with mutations occurring in different genes giving rise to very similar phenotypes or mutations in the same gene resulting in phenotypically different IRDs (3). Additionally, there appears to be both clinical and genetic overlap between some IRDs, such as Sorsby fundus dystrophy (SFD) and complex genetic disorders such as age-related macular degeneration (AMD) (4–6) These observations infer potential common pathomechanisms, not only between IRDs but also between IRDs and common complex retinal disorders. In particular, two key processes stand out, which contribute to cellular integrity, namely extracellular matrix (ECM) homeostasis and immunity. While much has been discovered in recent years about the immune response in retinal disease (7, 8), it is unclear if this occurs independently or whether it can be exacerbated by an altered ECM – the classic chicken-and-egg problem. A better understanding of the interplay between these two key processes and how they maintain a healthy retina may provide important information as to how aging and disease are impacted by their dysregulation.

The ECM is composed of structural macromolecules such as collagens, fibronectin, laminins, elastin and glycosaminoglycans (GAGs), as well as non-structural extracellular proteins (e.g., matrix metalloproteinases, MMPs; matrikines and matricellular proteins). These have been reported to affect trafficking, local concentration and the function of immune mediators (9, 10). Mechanistically, this is thought to involve an interaction between immune cells and molecules in the extracellular environment (10–12). Accordingly, areas of the eye enriched in ECM components, could represent dynamic multi-link interfaces critical for ocular immune response. In the first part of this article, we review the current understanding of the communication between the ECM and immune factors in the retina and their significance in maintaining homeostasis. We then discuss the regulatory role of matricellular proteins on retinal immune processes, how age and the extracellular environment may impact on retinal immunity and finally the role of the cellular microenvironment and inflammation in ocular disease.

## 2 The immunomodulatory role of ECM structural components in the retina

In the retina, structural ECM molecules can be found in basement membranes, the nerve fibre layer, the outer and inner plexiform layers, the interphotoreceptor matrix, and in Bruch’s membrane (BrM) (13). The latter is of particular interest as it represents a pentalaminar ECM structure between the choriocapillaris and the retinal pigment epithelium (RPE). It is formed by a dense core of elastic fibers surrounded by collagen-rich inner and outer layers, flanked by the basement membranes of the RPE and choriocapillaris endothelial cells. Structural elements are mainly glycosaminoglycans (heparan sulfate, chondroitin sulfate, and keratin sulfate), collagen I, III, V, fibronectin, and lipoproteins (14). In addition to its many functions as a structural support for the RPE and outer retinal vascular wall and as a transport pathway for fluids and macromolecules moving between the RPE and the choroid (14), BrM can also act as a modulator of innate immunity and retinal homeostasis. The pentalaminar structure of the BrM and the regular spacing of its ECM components enhances its molecular sieving capacity. It limits the passive diffusion of proteins with a molecular weight above 200 kDa from the choroidal blood supply to the RPE and vice versa (15). Moreover, BrM restricts the distribution of smaller molecules, such as components of the complement system (16, 17). In diffusion experiments with enriched BrM from human donors, Clark and colleagues showed that while some complement proteins (e.g., complement factor D, CFD; factor H-like protein 1, FHL-1; and C5a) diffuse through Bruch’s membrane, other central components such as C3 and C3b, complement factor B (CFB) and the complement inhibitors factor H (CFH) and factor I (CFI) are typically retained (18, 19). This could contribute to maintaining a reduced retinal inflammatory environment, as it physically separates systemic complement factors (along with those produced by the choroid) from those produced locally by the RPE and the neural retina (18).

Nonetheless, the concerted activity of the complement system at the choroid/BrM interface needs to be kept at bay to avoid damage to healthy tissue and subsequent excessive inflammatory responses. Here, CFH acts as the main negative regulator of complement activity, possessing both a cofactor activity for the breakdown of the central complement factor C3b and a cleavage activity against C3-convertase, responsible for the formation of C3 (20). By binding to GAGs, particularly to heparan sulphate (21), CFH and its splice variant FHL-1 protect the retina from inappropriate complement overactivation (19). As a consequence, the variability of heparan sulphate chains in terms of length, degree of sulphation and binding pattern to extracellular proteoglycans (21) are likely to influence the binding affinity of CFH, thus affecting its regulatory function (21). A modified binding of CFH could thus impair its inhibitory effect on complement, eventually leading to dysregulation of complement activity and consequent local chronic inflammation. In a pro-inflammatory milieu, immune cells such as neutrophils, B lymphocytes, monocytes, and platelets, which express receptors for CFH on their cell surface, are also attracted to the chemotactic properties of CFH (22–25). Interestingly, in a study by Schneider and colleagues, it was shown *in vitro* that CFH directly influences neutrophil activation, migration and spreading only when immobilized on a surface and not in solution (26). It is therefore conceivable that neutrophils may infiltrate a retinal lesion site as a first line of defense in a manner that depends on the composition of the ECM and the contact with CFH adhering to it.

**Figure 1:**
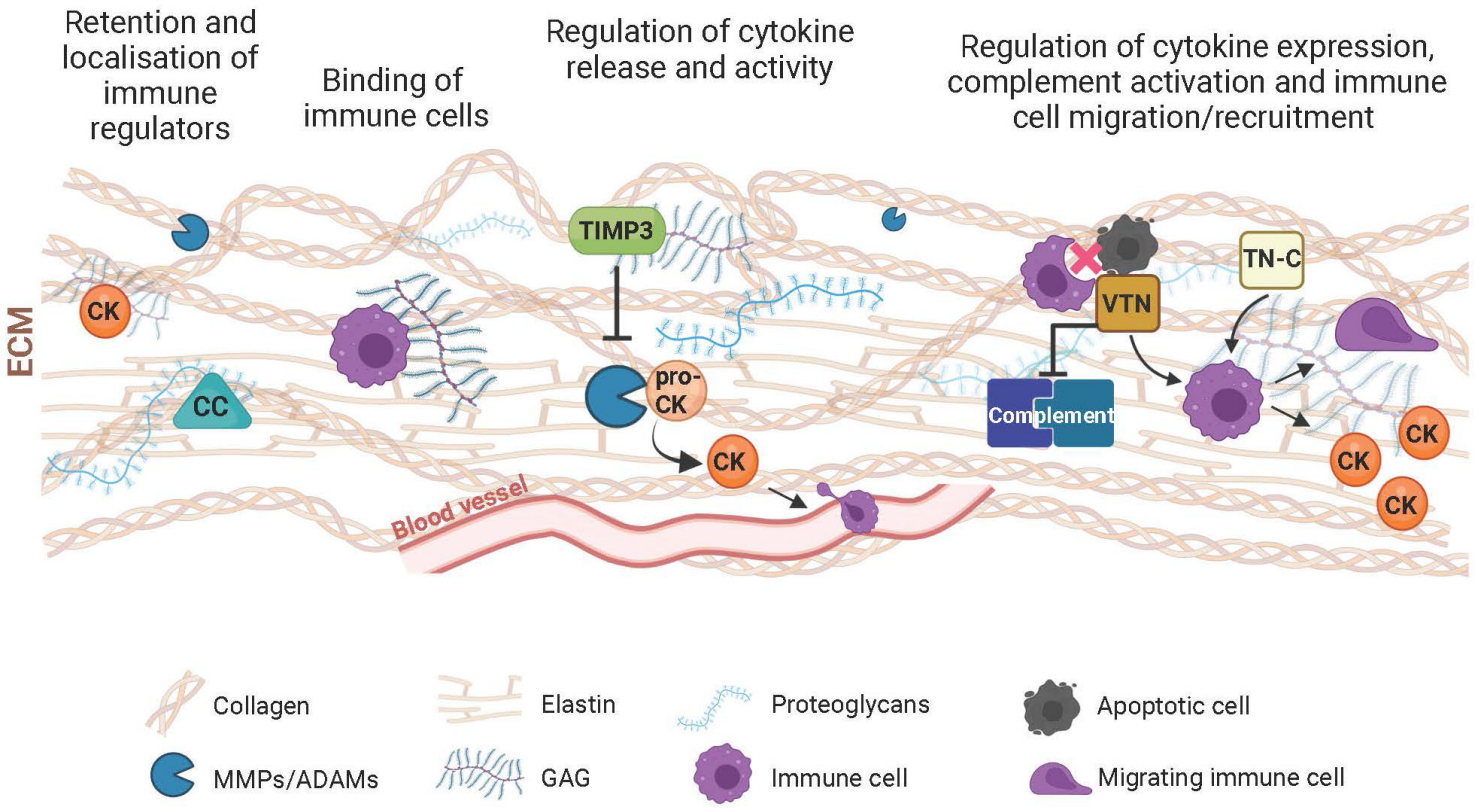
Schematic of components of the extracellular matrix (ECM) and their immunoregulatory role. The ECM participates in the control of the immune response with its structural and functional constituents through various mechanisms. Complement components (CC), cytokines/chemokines (CK) and immune cells are retained by the ECM meshwork or specifically bind to its structural components such as collagen, elastin, proteoglycans or glycosaminoglycans (GAG). This results in the local activity of immune regulators. Through turnover mechanisms involving extracellular proteases (e.g., MMPs or ADAMs), the ECM influences the transformation of proactive (pro-CK) to active CKs, thereby influencing their inflammatory and chemotactic function with consequent influence on the extravasation and migration of immune cells. Inhibitors of extracellular proteases, including the tissue inhibitor of metalloproteases 3 (TIMP3), further modulate immune regulation resulting from the digestion of the ECM and the inflammatory agents residing within. Matricellular proteins such as vitronectin (VTN) or tenascin-C (TN-C), which mediate the interaction between cells and their extracellular environment also influence ECM-associated immunological processes, including complement activation, immune cell migration, phagocytic removal of apoptotic cells, and secretion of inflammatory molecules.

In the ECM, GAGs also appear essential for the function of numerous inflammatory cytokines aimed at tissue growth and repair (27, 28). It is therefore plausible that appropriate abundance of GAGs distributed within the retina, particularly within BrM and the interphotoreceptor matrix (29)contributes to the binding of cytokines to the ECM. The resultant binding limits cytokine diffusion and the promotion of a localized activity of immune cells, including leucocytes (28). The recruitment of immune cells to the ECM is also modulated by the presence of hyaluronic acid, which binds to the CD44 adhesion receptor of leucocytes and macrophages (30). Collagen density may also directly modulate the immunoactivity of leukocytes (31) and macrophages (32). Therefore, its presence in the collagen-rich layers of BrM (but not in the interphotoreceptor matrix) can compartmentalize the migration and activity of immune cells in the retina. Furthermore, the collagen and elastin fibers of BrM may act as docking points for complement factors such as C3 and C4 (**Figure 1**). In support of this, covalent binding properties of these complement proteins for collagen and elastin fibers have been previously reported in vascular tissue (33).

Finally, it has been shown *in vitro* that changes in structural components of the ECM (e.g., fibronectin, collagen, laminin) deposited by RPE cells can in turn influence the deposition of immune factors, such as complement factors CFH and C3 (34). This supports the idea of a close relationship between structural components forming the retinal extracellular scaffold and immune defense pathways for homeostatic regulation which is highly dynamic in nature. Fragmentation, deposition and reorganization of ECM structural components are crucial for the maintenance of an optimal cellular microenvironment, as well as for native cell morphology and function, angiogenesis, and tissue repair. Metalloproteinases and their inhibitors contained in the ECM are also responsible for managing this dynamic process (35).

## 3 The contribution of matrix metalloproteinases and their inhibitors to retinal immunoregulation

Through their proteolytic action on most ECM components, MMPs (also known as matrixins) are crucial for the maintenance and homeostasis of tissues (36), including those of the retina (37). Moreover, MMPs have attracted increasing interest due to their ability to influence migration of immune cells, such as neutrophils and macrophages (38, 39), as well as the activity of inflammatory molecules such as cytokines and chemotactic derivatives (chemokines) by direct proteolysis (40, 41) or modification of their ECM substrates (42, 43) (**Figure 1**). This suggests an involvement of MMPs in the regulation of immune processes, such as leukocyte extravasation and infiltration and glial activation (44), and thus an influence on inflammation associated with various ocular disorders (45).

In the retina, the presence of various MMPs, mainly MMP-1, MMP-2, MMP-3 and MMP-9 have been identified in the interphotoreceptor matrix, BrM and the RPE (46, 47) and implicated in the regulation of the retinal immune response. For example, mice lacking MMP-3 are subject to acute lipopolysaccharide-induced inflammation, resulting in a reduced number of adherent leucocytes in the retinal vasculature compared to mice expressing MMP-3 (48).The absence of MMP-3 in the murine choroid/RPE complex resulted in reduced protein expression of the intercellular adhesion molecule-1 (ICAM-1), known to mediate leukocyte recruitment and adhesion during inflammation. Lipopolysaccharide treatment has been shown to lead to upregulation of inflammatory molecules (e.g., interleukin-6, tumor necrosis factor alpha, TNF-α; nitric oxide synthase) in the retina and RPE but absence of MMP-3 appears to inhibit this response (48).

Like MMP-3, MMP-9 can impact retinal homeostasis when its function as an immune enhancer involved in the processing, specificity, and receptor utilization of inflammatory molecules is altered (40, 49). This likely includes the potent pro-inflammatory interleukin 8 (IL8), which revealed a 10- to 30-fold increase in neutrophil activation following incubation and proteolytic cleavage by MMP-9 *in vitro* (40): In particular, neutrophils purified from human blood and incubated with the MMP-digested form of IL8 showed increased migration, activation of intracellular signaling and mobilization of intracellular calcium compared to cells exposed to the unprocessed form of IL8 (35). This suggests a sophisticated mechanism of regulation by extracellular proteases in the interaction between chemokines and their cognate receptors on neutrophils.

Since variations in IL8 production and/or activity are associated with the onset of inflammation and individual susceptibility to retinal diseases such as AMD (50, 51), the effect of MMP-9 on IL8 activity may have a direct influence on the pathogenic role of IL8 in the retina. In line with this, an increased intraocular concentration of both IL8 and MMP-9 has been measured in eyes of AMD patients and those with diabetic macular oedema (52, 53).

In an excellent review by Van Lint and Libert (54), the authors point out that processing of cytokines by MMPs can lead to their potentiation but also to their inactivation. After proteolytic cleavage, truncated cytokines can lose their activity, but they can also counteract the activity of intact cytokines by steric hindrance of the receptors on the surface of cells such as leucocytes. By doing this, they exert an anti-inflammatory effect (54, 55).

Furthermore, Starr and colleagues (41) demonstrated that MMPs can influence the binding affinity and release of chemokines such as chemokine (C-C motif) ligand 16 (CCL16) to and from GAGs in the ECM. This, in turn, influences the concentration of chemokines and their effect on migration, recruitment and activation of immune cells *in situ* (chemotactic potential) (41). As a consequence, cytokines further upregulate the cellular expression and production of MMPs. For example, cytokine-mediated upregulation of MMP expression has been demonstrated in cultured RPE cells (56). Following this line of argument, pharmacological inhibitors of MMPs have been suggested as a potential therapeutic agent to counteract inflammation in the retina (57–60). In particular, for those retinal diseases where upregulation/dysregulation of MMPs is known to occur, e.g., AMD and diabetic retinopathy (DR) (61–64).

*In vivo*, the activity of MMPs is inhibited by tissue inhibitors of metalloproteinases (TIMPs) (65). In human, the TIMP protein family comprises four members (TIMP-1 to 4). Of these, TIMP3 is the only one to be incorporated into the ECM due to its high affinity for proteoglycans (66). In the retina, TIMP3 is mainly expressed and secreted by the RPE and deposited in BrM (67, 68). There, it participates in regulating the degradation and turnover of structural constituents of the ECM, thus influencing the microenvironment outside the RPE and choroidal cells. In addition to its regulatory functions for MMPs, TIMP3 has also been reported to possess immunoregulatory properties.

In TIMP3-deficient mice the absence of TIMP3 coincided with an infiltration of neutrophils and macrophages in various organs, resulting in an increased inflammation (69–73). This is attributed to the ability of TIMP3 to inhibit the pro-inflammatory transduction signal induced by TNF-α, a pleiotropic cytokine produced predominantly by activated macrophages regulating apoptosis and the inflammatory response upon receptor binding (74). Specifically, TIMP3 inhibits the TNF-α converting enzyme (TACE/ADAM17), which transforms the membrane-bound proTNF-α into a soluble active form (75) (**Figure 1**). Inappropriate TNF-α production or prolonged activation of TNF-α signaling can predispose to a broad spectrum of inflammatory diseases, including retinal disorders such as glaucoma or DR (76, 77).

Treatment of cultured retinal microvascular endothelial cells with purified TIMP3 was further demonstrated to alter the expression of ICAM-1, which is a known regulator of immune cell transmigration. In addition, a TIMP3-dependent downregulation of cytokine-induced vascular adhesion molecule-1 (VCAM-1), which controls endothelial leukocyte adhesion and recruitment, and a reduction in vascular endothelial growth factor (VEGF)-induced migration and proliferation was also observed in these cells (78). Together, these results suggest a role for TIMP3 in preventing extracellular engagement of circulating anti-inflammatory molecules but also support the hypothesis of its anti-angiogenic effects, which limits the formation of new vessels that would otherwise potentially increase tissue inflammation (79, 80).

## 4 The immunological consequences of ECM fragmentation: The role of matrikines

During matrix remodeling, proteolysis of ECM structural proteins by extracellular enzymes such as MMPs can lead to the release of small, soluble, bioactive peptide fragments called matrikines. Compared to progenitor molecules, which play a predominantly structural role, matrikines regulate a range of cellular activities, including cell migration, proliferation, protease production, or apoptosis by binding directly to cell membrane receptors (81, 82). For example, the matrikine endostatin has long been known for its anti-angiogenic properties in homeostasis of the ocular vasculature and prevention of retinal neovascularization (83–85) as it inhibits endothelial cell migration and capillary morphogenesis (86, 87). Endostatin represents a cleavage product of collagen XVIII, associated with the basement membrane of almost all epithelia and endothelia, including the choriocapillary basement membrane of BrM (88–90). Liu and colleagues (91), in their work exploring the effect of endostatin on growth, angiogenesis and inflammation in mouse tumorigenesis, suggested a close relationship of this matrikine with the immune system. In particular, they observed a reduction in the expression of immunosuppressive cytokines (IL6, IL10, IL17, TGF-β) and a downregulation of inhibitory immune cells by endostatin. In contrast, an infiltration of immune dendritic cells and cytotoxic T lymphocytes into the tumor was observed following endostatin treatment (91). Although studies on the immune role of endostatin in the retina seem to be scarce, the data available reveal its immunoregulatory effect, which may also have an impact on the retinal microenvironment that merits investigation.

In addition to angiogenesis-regulatory activity, immunomodulatory function appears to be a property shared by many matrikines reported to direct inflammation in various inflammatory diseases, mainly due to their acquired chemoattractive properties (92–95). For example, fragments resulting from the degradation of fibronectin, a structural component of BrM (14) have been shown to have pro-inflammatory properties *in vitro*. Exposure of murine RPE to high doses of fibronectin fragments induced a significant increase in the RPE expression of the inflammatory cytokine IL6 and of the monocyte chemoattractant protein-1 (MCP-1), a key chemokine regulating the recruitment of monocytes/macrophages (96). Furthermore, fibronectin fragments induced increased expression of MMP-3 and MMP-9 in murine RPE cells. Based on findings that fibronectin expression by the RPE is increased in retinal disease development (97), Austin and colleagues discussed their results in the context of AMD pathogenesis, suggesting that the action of fibronectin fragments may contribute to a chronic RPE-mediated inflammation that may lead to abnormal BrM remodeling, deposit formation and drusenogenesis (96). Similarly, increased elastin-derived matrikine serum levels resulting from fragmentation of elastin in the ECM have been reported in AMD, particularly in the neovascular form, and discussed as potentially contributing to disease complications (98).

## 5 The regulatory effects of matricellular proteins on immune processes in the retina

Non-structural glycoproteins of the ECM meshwork that mediate cell-ECM communication are referred to as matricellular proteins. Interestingly, many of these proteins are found with low expression in healthy adult tissues, but increase in expression during inflammatory and pathological processes, suggesting their vital role in the immune response (99). This section addresses more closely the immune significance of two representative matricellular proteins namely vitronectin and tenascin-C.

### 5.1 Vitronectin

In basement membranes and the ECM, vitronectin is a multifunctional matricellular glycoprotein involved in the regulation of numerous processes, including cell adhesion and migration, matrix deposition and remodeling, fibrinolysis, angiogenesis, and coagulation (100, 101). The association of vitronectin with inflammatory diseases and its upregulation following inflammatory stimuli indicate an involvement of this protein in the immune response (102–109). Indeed, vitronectin has long been reported as a complement inhibitor (**Figure 1**) that binds complement proteins C5b-7 and C9 to prevent their attraction to the cell membrane. As a result, the membrane attack complex (MAC), which forms cytotoxic pores to drive cell lysis and inflammatory processes, no longer forms on target cells (101, 110).

In more recent years, the role of vitronectin in immune regulation has been extended to areas other than complement regulation. Most notably, vitronectin appears to mediate the recruitment of circulating inflammatory cells by promoting their passage through the endothelium (106, 108, 111, 112). Specifically, it has been shown that in complex with plasminogen activator inhibitor-1 (PAI-1), vitronectin is essential for the binding of neutrophils to microvascular endothelial cells, a crucial step in the immune response towards immune cell extravasation into the perivascular space and thus in inflamed tissues. *In vitro*nectin-deficient mice a drastic reduction in the adhesion of circulating neutrophils to postcapillary endothelial cells of the cremaster muscle subjected to inflammatory stimuli was observed (108). Such an effect of vitronectin was thought to be attributable to its ability to bridge interaction between endothelial cell basement membrane GAGs and the low-density lipoprotein receptor (LRP-1) bound by the vitronectin binding partner PAI-1 on the surface of circulating neutrophils. Upon carrying out their function of killing invading organisms or damaged cells, neutrophils undergo spontaneous apoptosis, preserving the integrity of the neutrophil membrane and preventing the release of toxic cytosolic cell contents from destroying surrounding tissues (113). At this point, macrophages intervene to resolve the inflammation by removing apoptotic neutrophils (a process known as efferocytosis) (113). Both *in vitro* and *in vivo*, vitronectin has been found to decrease neutrophil efferocytosis by binding to macrophages and apoptotic neutrophils through interaction with membrane integrins (αvβ3 and αvβ5) and the urokinase plasminogen activator receptor (uPAR), respectively (114). These results presented by Bae and colleagues support the hypothesis that vitronectin binding to different receptors on the two cell surfaces, may inhibit the receptors from interacting with each other directly or *via* bridging proteins, preventing recognition between macrophages and apoptotic cells and thus the latter from engulfment by macrophages (114) (**Figure 1**).

Notably, the binding of vitronectin to αv integrins on macrophages also induces nuclear transcription factor kappa B (NF-κB)-mediated expression of the inflammatory cytokines TNF-α, IL1β and IL6 (**Figure 1**), as demonstrated by experiments with bone marrow-derived murine macrophages (109). Vitronectin-dependent expression of IL6 and leukemia inhibitory factor (LIF) was also observed in cultured endothelial and glial cells as well as in mouse brains after treatment with recombinant vitronectin (115). An effect of vitronectin on microglial activation was also detected in a mouse model of experimental autoimmune encephalomyelitis. Immunohistochemical analysis revealed a link between perivascular deposits of vitronectin (as well as fibronectin) in the murine brain and the number of activated microglial cells (105). The investigations of Chakravarty and colleagues on the role of vitronectin in the atherosclerotic inflammatory process suggested an alternative mechanism. This attributes an increase in inflammation with the recruitment of the pro-inflammatory molecules NF-κB, ICAM-1 and VCAM-1 as a consequence of systemic vitronectin deficiency (116). These latter findings raise the possibility of vitronectin as a quencher or promoter of inflammation-related processes.

In the retina, the immunomodulatory properties of vitronectin have been scarcely studied. Vitronectin is produced by RPE cells, photoreceptors, and ganglion cells, but is found mainly in BrM and in the retinal vasculature (14, 117, 118). Studies on cultured immortalized RPE cells (ARPE-19) have shown that treating cells with commercial human complement serum (119) or inducing MAC formation (120) results in a significant increase *in vitro*nectin production. Given the inhibitory properties of vitronectin on complement, these results suggest that increased vitronectin production may defend RPE cells from uncontrolled complement activation (120). Consequently, vitronectin may mediate chronic low-grade inflammation in the retina, such as that arising with ageing (121). This could be related to the accumulation of vitronectin, along with other complement components, in drusen (sub-RPE and subretinal extracellular deposits) that have also been associated with the age-related manifestations in AMD pathogenesis (122, 123).

Finally, vitronectin may indirectly be involved in the immune regulation of the retina. We have previously shown that deposition of vitronectin in the ECM produced by ARPE-19 cells visibly affects the accumulation of structural matrix components such as collagen, fibronectin, and elastin (124). The altered production of vitronectin in the retina could therefore influence the immunoregulatory functions of the structural components of the ECM. Consequently, it can affect the exposure of neo-epitopes by the ECM in the binding of complement components or influence the accumulation of chemotactic proteolytic ECM fragments, as discussed above. Furthermore, we observed an increase in complement components C9 and CFH in the ECM of ARPE-19 cells containing vitronectin compared to the ECM lacking detectable amounts of this protein, which could further support the immunoregulatory role of vitronectin in the retina. Interestingly, the rs704 polymorphism in the vitronectin gene (*VTN*) has been associated with an increased risk of AMD (6). Studies have shown that this polymorphism leads to increased vitronectin protein expression (124, 125), which in turn could result in an exacerbation/dysregulation of its functional activities. Hypothetically, this could affect the immunoregulatory activities of vitronectin and contribute to retinal immune dysregulation, such as that associated with AMD (17, 126).

### 5.2 Tenascin-C

The matricellular protein tenascin-C is a ligand of toll-like receptor 4 (TLR4) and integrins (αvβ3 and α9β1) and is located on the membrane of several cell types, e.g., macrophages, dendritic cells and microglia. Its manifold immunoregulatory functions have been excellently summarized by Marzeda and Midwood (127). By binding to cell surface receptors, tenascin-C exerts mainly pro-inflammatory effects stimulating intracellular signaling towards the production of inflammatory cytokines (IL6, IL8, TNF-α), as well as the migration and polarization of immune cells, particularly in the context of neuroinflammation (**Figure 1**) (128). In contrast, an anti-inflammatory property was shown for tenascin-C conferred to either by itself or through its ability to bind and increase the local concentration of the immunosuppressive cytokine TGF-β. In mouse models of tenascin-C deficiency, the absence of this matricellular protein correlated mainly with altered immune cell infiltration and inflammatory response (127). For example, Manrique-Castano and colleagues demonstrated that, upon cerebral ischemia, tenascin-C-deficient mice exhibited reduced microglial coverage and increased leucocyte infiltration of the ischemic region compared to wildtype mice (129). Interestingly, the absence of tenascin-C seems to protect mice from the formation of amyloid deposits associated with Alzheimer disease and from experimental autoimmune encephalomyelitis (127).

In the retina, tenascin-C is predominantly expressed by amacrine and horizontal cells, thereby concentrating the matricellular protein in the outer and inner plexiform layers. Moreover, a considerable amount of tenascin-C is produced by astrocytes of the optic nerve (13, 130–132). The role of tenascin-C in retinal homeostasis is emerging through studies aimed at understanding pathogenic mechanisms such as the relationship between ECM remodeling and immunological processes in ocular diseases such as glaucoma, which is characterized by progressive retinal ganglion cell death and optic nerve damage (133), AMD, and DR (as reviewed by Reinhard and colleagues (134). In glaucoma, immune dysregulation is also discussed among potential causes that predispose to this disease (135, 136).

Wiemann and colleagues recently demonstrated that mouse models of experimental autoimmune glaucoma show differences in glial response and cytokine release depending on whether tenascin-C is expressed or not (137). Specifically, ganglion cell degeneration was induced in these mice by immunization with optic nerve homogenate antigens. Immunostaining and western blot analysis of retina and optic nerve sections showed an increase in astrocytes labelled with glial fibrillary acidic protein (GFAP) in the ganglion cell layer of wildtype mice, while tenascin C-deficient mice subjected to the same treatment showed a reduced microglial response. Likewise, tenascin C-deficient mice exhibited a reduction in Iba-1-positive microglial cells (137). Moreover, in the analysis of retinal and optic nerve mRNA expression, the absence of tenascin-C coincided with a reduction in the pro-inflammatory cytokine *TNF-α* and an increase in the anti-inflammatory cytokine *TGF-β* (137). In line with this and other studies (138–140), a dramatic upregulation of tenascin-C in murine retinal tissues of intraocular pressure-dependent glaucoma models has been described as part of an extensive remodeling of ECM molecules associated with glaucomatous neurodegeneration (141).

Tenascin-C upregulation has also been associated with ischemia-associated retinal degeneration (142) and vascular disorder associated-retinal diseases such DR (143–147) with the latter known to affect the retinal microvascular circulation (148). Tenascin-C has been described as increased in the basement membrane of diabetic retinal endothelial cells, where it is thought to influence ECM formation/remodeling and vascularization associated with DR, potentially interacting with other extracellular molecules such as fibronectin and periostin (145–147). Similarly, tenascin-C has been detected in choroidal neovascular membranes (CNVs) and BrM of patients with neovascular AMD (149–152). Here, it is thought to exert an anti-adhesive effect on RPE cells (152) and likely promotes neovascularization *via* integrin αv-mediated promotion of endothelial adhesion, migration, and neo-angiogenesis, as demonstrated *in vitro* with cultured endothelial cells (human dermal microvascular endothelial cells, HMVECs) (151). In line with this, tenascin C-deficient mice showed a significant reduction in the extent of choroidal vascularization (151).

It remains to be determined whether tenascin-C and its angiogenic function play a significant role in the immune dysregulation and inflammation that affects the aforementioned retinal diseases such as DR and AMD. The stimulation of tenascin-C production by retinal endothelial cells through inflammatory cytokines TNF-α and IL1β does appear to suggest a potential involvement of this matricellular protein in the immune response in both homeostasis and diseases of the retinal endothelium (146).

## 6 Age-related ECM changes further point to a link between the extracellular environment and retinal immunity

The function of the ECM and its structural and non-structural constituents in modulating immune responses can be drastically impaired by age-dependent changes in the extracellular environment. As extensively reviewed by Moreau and colleagues (153),age-related changes in the ECM, including increased cross-linking and stiffness of collagen, would be a major contributor to so-called immunosenescence, an age-related loss of immune efficacy. These changes have been reported to primarily affect the mobility of immune cells, particularly tissue-resident lymphocytes, and neutrophils, which is essential for their effective mediated immunity. It has been discussed that the movement of these cells within a much stiffer matrix due to ageing causes damage to the cell membrane and nucleus, ultimately leading to immune cell death and immunodeficiency (153). In contrast, neutrophils seeded *in vitro* on very dense and rigid matrices showed increased activation in terms of formation of neutrophil extracellular traps, a network of DNA/histone complexes and proteins with pro-inflammatory and microbiocidal effects. Such effects are enhanced by the presence of ECM proteins, including fibronectin and vitronectin, which mediate the activation of focal adhesion kinase upon integrin-mediated neutrophil interaction (154).

*In vitro*, macrophage immune function was also found to be sensitive to microenvironmental changes. In particular, culturing macrophages on stiffer matrices alone was sufficient to increase cell spreading as a result of the increased presence of integrin adhesion receptors, protruding filopodia and denser F-actin organization, as well as differentiation towards an anti-inflammatory phenotype with pro-tissue repair functions (155–159). These include the secretion of cytokines (IL10, IL1β), chemokines and growth factors (VEGF, TGF-β), as well as ECM remodeling proteases, which further modify the content and turnover of the ECM (160).

Similarly, Wong et al. showed that mesenchymal stromal cells respond to changes in matrix stiffness with cytoskeletal changes and surface receptor reorganization (161). In contrast to rigid matrices, in soft matrices these cells reacted with actin polymerization and clustering of TNF-α receptors resulting in increased TNF-α binding and, in turn, activation of TNF-α-induced signaling pathways that are likely implicated in the production, differentiation, and recruitment of leukocytes (161). The adhesion of the latter to endothelial cells, necessary for their extravasation into perivascular tissues, also appears to be affected by matrix stiffness (162, 163). With aging, hardening of the subendothelial matrix leads to an increase in the permeability of the endothelial monolayer due to increased cellular contractility and loss of cellular junctions. This in turn could promote increased leukocyte transmigration (162).

The retinal ECM undergoes reorganization with aging. For example, BrM develops a significant stiffening and thickening due to the accumulation of lipids, GAGs, collagen, and elastin (14). This may have implications for immune cell activation and function in the immune response. In addition, due to its thickening, BrM shows an age-dependent decrease in permeability (14), possibly leading to abnormalities in the compartmentalization of complement factors. *In vitro*, aging of BrM is simulated by nitrite modification of the ECM produced by RPE cells in culture. The growth of RPE cells on this type of matrix appears to influence the secretome of RPE cells. Indeed, these cells were found to secrete significantly higher levels of complement factors such as C3a than cells grown on unaged matrices (164). The effect of nitrite-modified matrices on complement gene expression was also evident in RPE derived from human induced-pluripotent stem cells (iPSCs) from AMD patients. When these cells were grown on nitrite-modified matrices a significantly increased expression of complement immune genes such as *C3*, *CFB* and *CFH* was observed when compared with age-matched controls (165). This suggests that changes in the ECM may strongly contribute to or underlie an altered/increased immune response in retinal diseases such as AMD. Based on such findings, the reduced collagen solubility and loss of elastin in BrM associated with aging (14) can potentially affect immune cell recruitment and activity, binding and activation of complement factors, and propagation of inflammatory cytokines. In line with this, Kenan and colleagues found a reduced amount of heparan sulfate in BrM and neurosensory retina in macular tissue sections from older probands compared to younger donors (166). Again, the results point to a role of alterations in ECM components impacting on the immune response associated with retinal aging and thus susceptibility to disease.

It should be noted that in ageing BrM, increased binding of extracellular MMPs to the ECM appears to underlie their sequestration and thus reduction of proteolytic potential (47, 167). In addition, this impacts the remodeling and composition of the ECM, resulting in reduction in the degradative capacity of MMPs in aged BrM and subsequent consequences for the release, enhancement, or inactivation of cytokines. Finally, age-dependent alterations in the expression and secretion of matricellular proteins may also contribute to changes in the extracellular environment with aging. By analyzing the expression of the *VTN* gene in a large-scale collection of 311 healthy retinal tissue samples, we found a statistically significant increase in the expression of this matricellular protein in retinal samples aged 60 years or older (168). This implies an increased accumulation of vitronectin in retinal extracellular spaces, such as BrM, with consequences for its immunoregulatory functions.

## 7 The cellular microenvironment as a site of origin of immune dysfunction and inflammation in ocular disease

Dystrophies of the retina and the RPE represent broad clinical entities with many sharing a number of clinical features often rendering differential diagnosis demanding. Mutations in more than 300 different genes have been reported to be associated with degenerative changes in the retina and the RPE (169), although so far, causal genetic variants can be identified in only about 40-70% of all retinal patients. Many retinal dystrophies are monogenic in nature, i.e., they are caused by defined mutations with strong effect sizes in a single gene. In contrast, age-related macular degeneration (AMD), the third most common cause of legal blindness globally is a complex disorder, where disease risk is influenced by both genetic and environmental factors (170, 171). While causes and phenotypic presentations may greatly vary within the group of retinal conditions, a growing number are recognized to involve the ECM and a dysregulation of the immune response.

**Figure 2:**
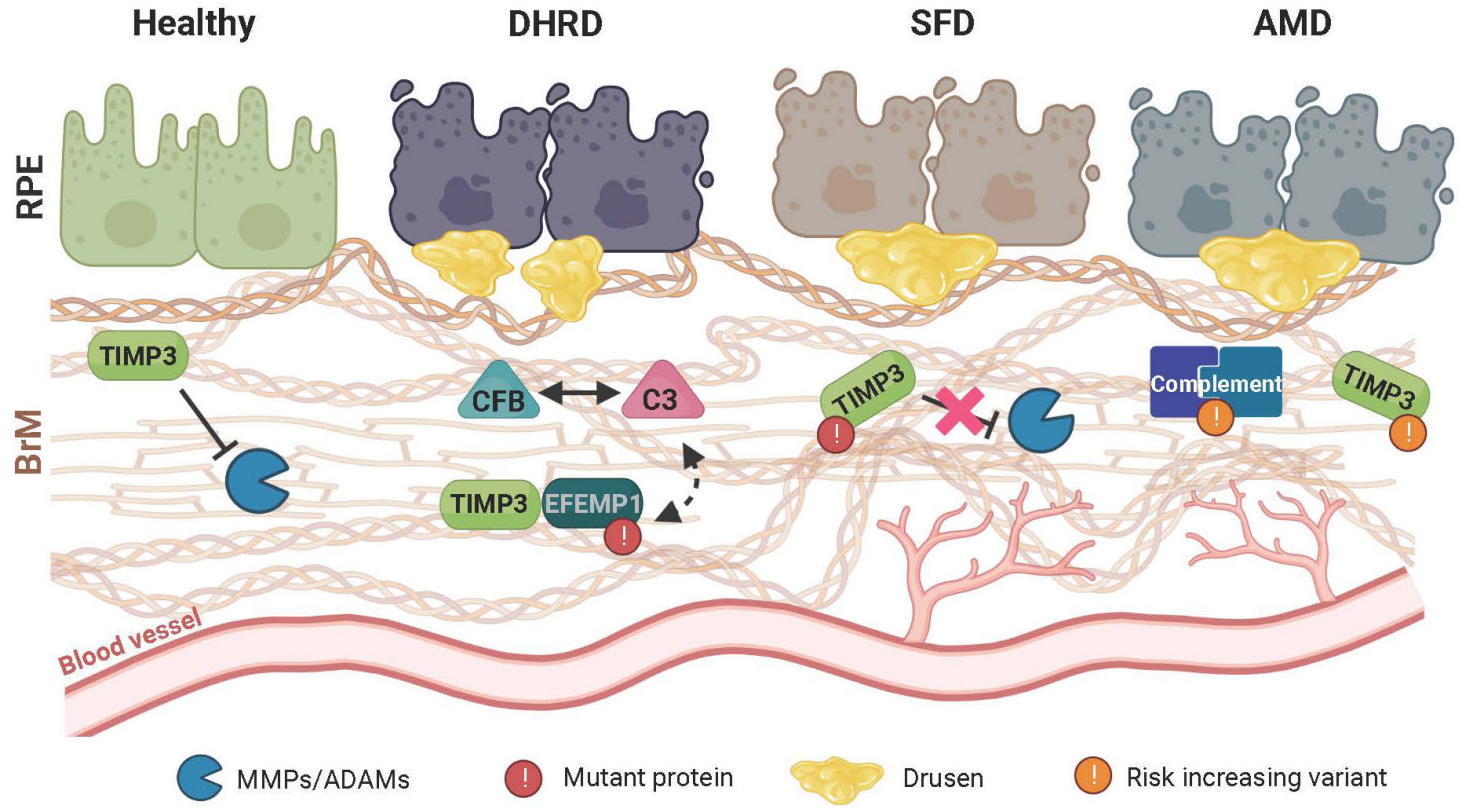
Schematic representation of retinal disease pathology on extracellular matrix (ECM) homeostasis and immune regulation. In the healthy retina, a finely tuned balance maintains the structural integrity of the extracellular Bruch’s membrane (BrM) to prevent inflammation and sustain a beneficial cellular environment for the retinal pigment epithelium (RPE) and the underlying choroidal blood vessels. Remodeling of BrM by MMPs and their regulation through TIMP3 plays an important role in maintaining this homeostasis. In Doyne honeycomb retinal dystrophy (DHRD), which is caused by a single mutation in the epidermal growth factor-containing fibulin-like extracellular matrix protein 1 (*EFEMP1*) gene, an interplay between ECM remodeling and an aberrant activation of the complement system e.g., due to a concomitant increased protein expression of complement component 3 (C3) and complement factor B (CFB), leads to structural changes in BrM and formation of drusen-like deposits, ultimately resulting in RPE atrophy. In Sorsby fundus dystrophy (SFD), which is caused by mutations in the TIMP metallopeptidase inhibitor3 (*TIMP3*) gene, ECM remodeling and BrM homeostasis is altered leading to RPE atrophy. Subsequently, drusen deposition ensues, which is often accompanied by formation and invasion of new blood vessels towards the RPE. While pathological changes in DHRD and SDF are caused by mutations in single genes, age-related macular degeneration (AMD) is a common disease involving many genetic risk variants with a complex interplay of risk increasing alleles in several components of the complement cascade as well as ECM remodeling enzymes such as TIMP3. Of note, pathological changes seen in AMD closely resemble those seen in SFD.

The following section provides an overview on the involvement of ECM homeostasis and immune responses in the pathological processes of both monogenic and complex retinal disorders (**Figure 2**).

### 7.1 Doyne honeycomb retinal dystrophy

DHRD, also known as Malattia Leventinese (ML), is an example of a rare autosomal dominant monogenic disorder caused by a single mutation, namely the R345W missense mutation in the gene encoding the epidermal growth factor-containing fibulin-like extracellular matrix protein 1 (EFEMP1), an extracellular glycoprotein also known as fibulin-3 (172). DHRD/ML patients do not usually become symptomatic until after age 30 with clinical characteristics of the disease including macular sub-RPE deposits/drusen and RPE degeneration which in the advanced stages can also be associated with choroidal neovascular membranes and geographic atrophy.

The molecular pathomechanism in DHRD/ML remains elusive, although the EFEMP1 R345W mutation is speculated to result in increased protein misfolding which may lead to disturbances in the proteostasis network resulting in protein aggregation and drusen formation (173, 174). Molecular constituents of drusen in DHRD/ML include vitronectin, amyloid P, TIMP3 and a number of complement proteins, all components also found in drusen of AMD patients (175, 176). Animal models have greatly fostered our knowledge about the molecular pathomechanism of DHRD/ML disease. *Efemp1* ^R345W/R345W^ knock-in mice mimic the phenotype seen in human patients and show formation of sub-RPE deposits which do not form in wildtype littermates or *Efemp1^-/-^* knock-out mice of the same age (177). In fact, *Efemp1-*deficient*^-^* mice intriguingly appear to be protected from drusen formation, suggesting a role of murine Efemp1 in generating the drusen-like deposits (178). Early work applying proteomic analyses of BrM in *Efemp1* ^R345W/R345W^ mice indicated that basal deposits contain normal ECM components, but these are present in abnormal quantities. These studies further revealed significant changes in proteins with immune related function, including those of the complement cascade as part of the immune system (175). Additionally, cultured primary RPE cells from *Efemp1* ^R345W/R345W^ mice have been shown to secrete increased amounts of complement factor 3 (C3) (179). Together, these findings further strengthen the notion that the immune system and inflammation are associated with drusen formation.

To clarify the mutual interplay between changes in ECM/BrM and inflammation, three double-mutant mouse lines were generated including an *Efemp1* ^R345W/R345W^/*C3^-/-^* double mutant mouse (175). The authors demonstrated the requirement of C3 for drusenogenesis, as the double-mutant animals failed to produce sub-RPE deposits. Such findings support the idea that the complement system plays a key role in basal deposit formation, likely through recognition of abnormal ECM. C3 is a key player in the complement system, as it is the first node where the three different upstream activation mechanisms (the alterative, classical and lectin pathways) converge and from thereon follow an identical downstream pathway ultimately culminating in MAC formation.

By analyzing *Efemp1* ^R345W/R345W/^ *C5^-/-^* double mutant mice, Garland and colleagues aimed to elucidate whether C3 rather than C5 function is required for drusen formation in DHRD/ML. They demonstrated that genetic ablation of *C5* did not prevent the formation of sub-RPE deposits, but rather increased drusen formation in the mutant animals (175). In *ex vivo* cell cultures strong immunostaining for C3 was demonstrated in RPE cells from *Efemp1* ^R345W/R345W^ compared to RPE cells derived from wildtype animals. In addition, Efemp1 protein colocalized with murine Cfh in the sub-RPE deposits. Based on their results, Garland and colleagues proposed that increased activation of C3 occurs in *Efemp1* ^R345W/R345W^ mediated by the alternative complement pathway activation mechanism of tick-over *via* the deposition of C3b on abnormal ECM (180). It remains to be shown whether mutant Efemp1 causes presentation of ligands that ultimately activate the classical pathway.

Extending these initial studies, additional data were presented recently demonstrating that complement factor B is critical for sub-RPE deposit formation in the *Efemp1* ^R345W/R345W^ mouse model (177). Quantitative RNA sequencing and subsequent proteomics demonstrated that expression of inflammatory pathway genes was increased in neural retina and posterior eyecups in 17-month-old *Efemp1* ^R345W/R345W^ mice compared to wildtype mice. *Efemp1* ^R345W/R345W/^ *Cfb^-/-^* double mutant mice revealed partial restoration of the changes that were seen in *Efemp1* ^R345W/R345W^ single mutant animals, suggesting a key role for murine Cfb in sub-RPE deposit formation. This was further supported by applying a small molecule inhibitor of Cfb reducing sub-RPE deposition by 65%. This reduction, however, was only observed in female mice, but not in male animals (177). Interestingly, loss-of-function alleles in the *CFB* gene in humans are associated with a decreased risk of developing AMD (181, 182).

In summary, various animal studies suggest that mutant Efemp1 protein leads to activation of the alternative complement pathway, which then drives the formation of sub-RPE deposits. In addition to mouse models, patient-derived cell lines, such as iPSCs-derived RPE cells have become a valuable model to study DRHD/ML. iPSC-RPE cells harboring the *EFEMP1* R345W mutation show an increased number of drusen-like deposits compared to controls after only 90 days of cultivation on transwell filters. Additionally, mRNA expression of complement genes *C5* and *MCP* is significantly upregulated in DHRD/ML cells and *CFB* was also shown to be elevated but failed to reach statistical significance (183).

### 7.2 Sorsby fundus dystrophy

DHRD/ML not only shares striking phenotypic similarities with AMD but also with the seemingly unrelated SFD. The latter monogenic maculopathy was first described by Arnold Sorsby and colleagues in 1949 (4) and was reported to ultimately result in bilateral loss of central vision. SFD is inherited in an autosomal dominant mode and is classified as a rare disease with a prevalence of 1:220,000. In contrast to AMD, the onset of SFD is much earlier between the second and fourth decade of life (4). Early symptoms of SFD include nyctalopia (night blindness) or loss of visual acuity, with later complications such as oedema, hemorrhages and macular scaring. Further progression of the disease culminates in retinal and choroidal atrophy (chorioretinal atrophy) and vessel sclerosis, which leads to severe central and peripheral vision loss about 35 years after disease onset (4, 184). In contrast to DHRD/ML with only a single mutation in *EFEMP1*, a growing number of independent genetic variants in the *TIMP3* gene are causative for SFD. Most of these variants result in an uneven number of cysteine residues in the mature TIMP3 protein. In healthy eyes, TIMP3 is expressed by the RPE and accumulates in BrM (5). A major role for TIMP3 relates to its involvement in the regulation of BrM thickness (185). Mutant TIMP3 protein is suggested to be more resistant to turnover and clearance in BrM, which ultimately results in accumulation of TIMP3 in drusen-like deposits (186–189). An increasing thickening of BrM results in impaired exchange of nutrients and molecules between RPE cells and the choroid and finally leads to RPE atrophy (185, 187).

Enhanced RPE autofluorescence due to oxidation products of unsaturated fatty acids is caused by the intracellular accumulation of lipofuscin, which is often referred to as a “wear-and-tear” pigment (190). Increased lipofuscin content of RPE cells is a normal feature of aging cells but has been linked to decreased lysosomal activity and promotion of local retinal inflammation (191–193). Activation of the complement system and secretion of pro-inflammatory cytokines appear to be a consequence of lipofuscin accumulation in the RPE. These findings indirectly link TIMP3 malfunction to immune responses as a consequence of BrM thickening. TIMP3 has also been implicated in the regulation of inflammatory processes in the diseased retina in a more direct manner. As mentioned above, TIMP3 not only inhibits MMPs, but also proteins of the ADAM and ADAMTS families. Specifically, ADAM17, which is also known as TACE (TNF-a-converting enzyme) is known to be directly inhibited by TIMP3 binding (194). ADAM17 is responsible for the cleavage of inactive pro-TNFα to its active form and hence plays a crucial role in TNFα-mediated signaling. This is further supported by the fact that a pronounced inflammation of the liver accompanied by increased levels of both TACE and TNFa was observed in Timp3 knockout mice (69). TIMP3 functions in the retina and other tissues have been recently reviewed (195). Further evidence for an involvement of TIMP3 in immunity and inflammation was obtained from studies in iPSC-RPE cells. iPSC-RPE cells derived from SFD patients showed increased levels of complement genes *C1R*, *C1S*, *C3*, *MCP* and *SERPING1* (183).

### 7.3 Age-related macular degeneration

In contrast to DHRD/ML and SFD, AMD is a complex disorder caused by both environmental and genetic factors. While monogenic disorders are typically caused by mutations in a single gene, the genetic susceptibility to complex disorders is generally influenced by genetic variants at multiple loci. In the largest genome-wide association study of AMD to date 52 variants in 34 loci were identified as being significantly associated with the disease at the genome wide significance level (6). Of note, 19 of these variants are located within or in proximity to genes coding for components of the complement cascade whereby the most striking association is seen with components of the alternative pathway. Both the highly significant enrichment of complement genes and the fact that several complement components have been found in drusen from AMD patient donor eyes (196–198) suggest an important role of complement dysregulation in AMD pathobiology. As an in-depth discussion of the complement system and its role in AMD pathogenesis is beyond the scope of this article, the reader is referred to two excellent recent reviews on this topic (199, 200). Another cellular feature which shows a marked enrichment in the 34 AMD associated loci is ECM remodeling and homeostasis, again linking the two topics of ECM homeostasis and immune regulation. Although the detailed interplay between inflammatory processes, drusen formation and disturbances in BrM homeostasis remains elusive at present, recent research, as discussed in the next paragraph, has proposed some hypotheses as to how these two key features of cellular maintenance may influence each other.

Not only in disease but also in physiological aging, BrM undergoes structural changes including increased thickness, altered permeability and deposit formation (drusen, hard and soft, basal linear (BlinD) and laminar (BlamD) deposits). To mimic an aged BrM, the use of non-enzymatically crosslinked nitrite-modified ECM has been explored *in vitro* (165, 201, 202). This is a particularly suitable model to study molecular pathomechanisms in AMD, as cigarette smoking leads to an increase in nitric oxide and represents a key environmental risk factor for the disease (203). Using ARPE-19 cells Fields and colleagues demonstrated that an altered ECM structure modifies the expression of CD46, which is a negative regulator of the complement pathway (202). A progressive decrease in CD46 has also been observed in RPE from GA patients, while Cd46 knock-out mice were shown to be more susceptible to CNV, likely due to impaired complement inhibition (204, 205). The same group confirmed their initial findings in iPSC-RPE grown on nitrite-modified ECM by demonstrating increased levels of bioactive C3a in the conditioned media of the cells (164). Another *in vitro* model for diseased BrM/ECM interestingly is based on the DHRD/ML-causing R345W variant of EFEMP1. Expressing the EFEMP-R345W variant in ARPE-19 cells caused the production of ECM with altered properties compared to cells expressing normal EFEMP1 (34). Due to the overlapping pathophysiological features of DHRD/ML and AMD, using this altered ECM has become an established system to mimic “diseased” ECM. These alterations can be attributed to increased MMP2 activation in cells expressing the R345W variant of EFEMP1. Analysis of complement components deposited in the altered ECM revealed increased C3b and CFH. When primary human RPE cells were cultivated on the altered ECM, they showed a significantly lower transepithelial electrical resistance and prominent basal deposits after 2 weeks in culture (34). These findings were subsequently corroborated by extending the model with iPSC-RPE cells which harbor different *EFEMP1* genotypes. Like primary RPE, iPSC-RPE showed an unwanted activation of the complement cascade when cultured on EFEMP1-R345W ECM derived from ARPE-19 cells. By using a sophisticated assay design and combinations of EFPEP1 and C3 single and double mutant iPSC-RPE lines it was shown that complement activation *via* the mechanism of tick-over is sufficient to induce basal deposit formation. Additionally, a siRNA knockdown of *C3* suppresses the formation of abnormal ECM, which corroborates the data obtained for deposit formation in DHRD/ML (206). Interestingly, an effect of C3a on ECM structure was also reported. Stimulation of human fetal RPE with C3a for two weeks resulted in an increased basal accumulation of collagens IV and VI mirroring ECM alteration, decreased proteasome activity and increased MMP-2 activity (34). While these models used EFEMP1 indirectly to mirror AMD-like pathological changes *in vitro*, there actually is also a more direct link between the EFEMP1 protein and AMD pathobiology. One of the genetic loci with the strongest association to an increased AMD risk at 10q26 comprises the gene *HTRA1* (*High Temperature Requirement A Serine Peptidase 1*). HTRA1 is a secreted protease which is implicated in a number of biological processes including cell signaling, organization of the ECM, and skeletal development and osteogenesis. One noteworthy target to be cleaved by HTRA1 is EFEMP1 (207). Even though there is no direct link between EFEMP1 and AMD pathogenesis, this interaction between HTRA1 and EFEMP1 yet again points towards a common disease mechanism possibly shared between different retinal dystrophies.

Taken together the findings gathered through research on different, yet similar pathologies, one could propose a model for the order of events in drusenogenesis as follows: in aging or disease, alterations in the structure of the ECM, or in particular in BrM occur, which then leads to the activation of the complement cascade which in turn initiates drusen formation. This order of events would also be in line with a model on drusen formation proposed by Anderson and colleagues who suggest that cellular debris can trigger an inflammatory signal, and which then gives rise to a potential “nucleation” site for drusen formation (208).

### 7.4 Diabetic retinopathy

DR is the leading cause of vision loss in the adult population and arises as a comorbidity of diabetes (209). Even though GWAS have identified a few hundred risk loci for diabetes, the contribution of the overall genetic risk to disease development is less clear than for example in AMD (210). A potential dialogue between ECM changes and immune responses has also been highlighted in the progression of DR, which results in visual impairment due to altered retinal vascular structure and macular oedema in hyperglycemic conditions. While the aforementioned diseases of DHRD, SFD and AMD primarily result in disturbances of BrM and the RPE, DR most prominently affects the retinal vasculature and to our knowledge, there is no evidence of altered BrM in DR. While the RPE may contribute to DR pathogenesis, it is not clear if it is the primary site of pathology in DR and more evidence is required (211). Recent advances in our understanding of ECM remodeling and inflammation in DR have excellently been summarized in two recent review articles (212, 213). We will therefore only touch upon some of the most current literature on implications of immune dysregulation and ECM alterations in DR.

In the last years convincing evidence has been gathered suggesting that DR represents a neurodegenerative disorder in its early stages. Retinal Müller glia cells are of outmost importance for retinal health and it has become apparent that they also play a role in DR (214). Using a diabetic pig model Sagmeister and colleagues conducted an in-depth proteomic analysis of changes induced in Müller cells under high glucose conditions (215). Interestingly, the majority of proteins found to be differentially expressed were proteins of the ECM. These findings are in line with data from many others, suggesting that alterations of the ECM are a very early events in DR pathogenesis. In DR, capillary ECM becomes fibrotic and is characterized by altered collagen IV structure as well as fibronectin and tenascin-C deposition. Functional consequences of altered ECM structure and composition observed in cell cultures under high glucose conditions include increased stiffness and higher permeability (213). While it is broadly accepted that ECM alterations occur in DR, it remains to be elucidated which molecular pathways trigger the ECM remodeling process. To deepen our understanding of this topic, Giblin and colleagues conducted a study in which they subjected human retinal microvascular endothelial cells (hRMEC) and human pericytes (hPR) to hyperglycemia, dyslipidemia and inflammation. These conditions are considered to represent three major insults in diabetic conditions. Of note, the most prominent and consistent changes in ECM expression were seen upon treatment with cytokines TNF-α and IL1β. The authors further showed, that treating hRMEC with conditioned media from IL1β treated Müller cells caused a marked increase in collagen IV and agrin expression (216)

As already mentioned, the interplay between ECM homeostasis and inflammation is by no means a one-way road, and while inflammation can result in ECM alterations, ECM-related processes can also regulate inflammation. In the context of DR, this was nicely demonstrated by a recent study by Abu El-Asrar and colleagues who suggest that intravitreal administration of TIMP3 in a DR rat model alleviates retinal inflammation by stimulating anti-inflammatory events (78). These events include reduced retinal vascular permeability and an attenuation of TNF-α-dependent retinal expression of the pro-inflammatory NF-κB and the adhesion molecule ICAM-1. Of note, this study also included Müller cells, in which treatment with TIMP3 counteracted induced upregulation of VEGF under various experimental conditions mimicking a diabetic environment. These findings once more highlight the manifold functions of TIMP3 in retinal homeostasis.

## 8 Conclusion, perspective and future directions - TIMP3 at the nexus of monogenic and complex diseases

**Figure 3:**
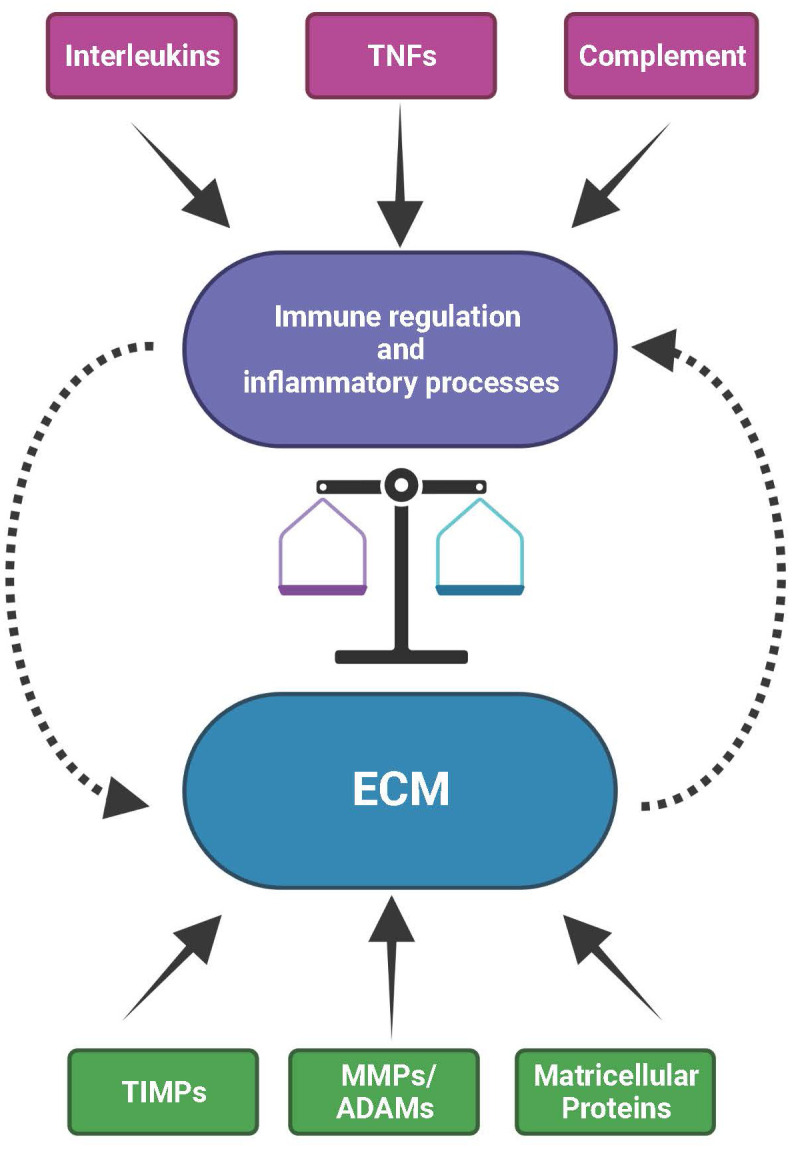
The mutual interplay between the extracellular matrix (ECM) and immunological processes. The ECM with its structural constituents but also its functional components, such as ECM remodeling enzymes (MMPs, ADAMs and TIMPs) and matricellular proteins, provides the microenvironment for the activity of immune and inflammatory processes. Vice versa, immunological/inflammatory activities contributed by e.g., interleukins, tumor necrosis factors (TNFs) or complement can influence the structural and functional organization of the ECM. As a consequence, disturbances in the expression or activity of factors that are part of the ECM or of the immune system affect the balance between inflammatory events and ECM homeostasis. These can eventually impact natural ageing, environmental insult or high genetic risk, together eventually initiating and sustaining onset and progression of progressive disease processes in the retina.

Monogenic IRDs such as DHRD/ML or SFD overlap and share several (histo)pathological and genetic features with more complex retinal diseases like AMD. In monogenic IRDs, great efforts have gone into gaining knowledge of the role of disease-causing genetic variation using both *in vivo* animal as well as *in vitro* cell culture models. The findings from such models may thus offer avenues for transfer to more complex retinal disorders. Here, we have summarized current knowledge on the commonalities between the divergent clinical conditions and their underlying pathogenicities demonstrating that alteration in the ECM/BrM turnover likely initiates or sustains similar immune responses in both monogenic and complex diseases (**Figure 3**). This insight may help to broaden our approaches in addressing the modulation of disease mechanisms and may potentially lead to novel designs in therapy of these devastating ocular diseases.

Of note, TIMP3 could highlight a nexus between monogenic and complex diseases with genetic variants in this gene not only being causative for the autosomal dominant SFD but also with non-coding variants in proximity of the gene being significantly associated with risk of AMD (6). Mutant TIMP3 protein is thought to assemble into pathologic aggregates in the SFD condition, very similar to AMD. In the latter situation, the aggregation is not caused by mutated protein, but is likely due to risk-dependent increased expression and intramolecular cross-linking of normal TIMP3 upon oxidative stress or due to spontaneous misfolding. This alternate route to protein aggregation takes an extended time to form, which may explain why AMD has a later onset of symptoms than SFD (185). Additionally, TIMP3 may also play a role in DHRD/ML as it is a direct interaction partner of EFEMP1 (217). Curiously, EFEMP1 also interacts with CFH (218), although there are currently no hypotheses as to how a potential interactome of ECM modulators and complement components may participate in the maintenance of BrM homeostasis. Finally, experimental evidence also supports the role of TIMP3 in ameliorating the inflammation, angiogenesis and vascular damage associated with DR (78).

Observations of commonalities between different clinical pictures may provide a unique opportunity to provide not only an improved model of disease but also to understand retinal pathomechanisms, particularly in complex diseases such as AMD. In light of such a reasoning, targeting the ECM or its molecular constituents could provide an alternative but yet important avenue for therapeutic intervention in retinal diseases in addition to those currently being explored through isolated immune targets. Such an approach appears superior as a common pathway can be addressed regardless of many individual mutational events or risk predispositions.

## Author contributions

KP, FB, PB, and BW were all involved in the concept, design, writing, and critical review of the content of this article. All authors agree to the accuracy and integrity of this work as presented. All authors contributed to the article and approved the submitted version.
